# Rac and Rho GTPases in cancer cell motility control

**DOI:** 10.1186/1478-811X-8-23

**Published:** 2010-09-07

**Authors:** Matteo Parri, Paola Chiarugi

**Affiliations:** 1 Externautics - R&D, via Fiorentina 1, Siena, SI, 53100, Italy; 2 Department of Biochemical Sciences, University of Florence, Tuscany Tumor Institute and "Center for Research, Transfer and High Education DenoTHE", 50134 Florence, Italy

## Abstract

Rho GTPases represent a family of small GTP-binding proteins involved in cell cytoskeleton organization, migration, transcription, and proliferation. A common theme of these processes is a dynamic reorganization of actin cytoskeleton which has now emerged as a major switch control mainly carried out by Rho and Rac GTPase subfamilies, playing an acknowledged role in adaptation of cell motility to the microenvironment. Cells exhibit three distinct modes of migration when invading the 3 D environment. Collective motility leads to movement of cohorts of cells which maintain the adherens junctions and move by photolytic degradation of matrix barriers. Single cell mesenchymal-type movement is characterized by an elongated cellular shape and again requires extracellular proteolysis and integrin engagement. In addition it depends on Rac1-mediated cell polarization and lamellipodia formation. Conversely, in amoeboid movement cells have a rounded morphology, the movement is independent from proteases but requires high Rho GTPase to drive elevated levels of actomyosin contractility. These two modes of cell movement are interconvertible and several moving cells, including tumor cells, show an high degree of plasticity in motility styles shifting *ad hoc *between mesenchymal or amoeboid movements. This review will focus on the role of Rac and Rho small GTPases in cell motility and in the complex relationship driving the reciprocal control between Rac and Rho granting for the opportunistic motile behaviour of aggressive cancer cells. In addition we analyse the role of these GTPases in cancer progression and metastatic dissemination.

## Review

### Rho and Rac GTPases

Rho proteins belong to the Ras superfamily. They are small (21-25 kDa) molecules that share structural homology and become activated only when bound to GTP. The best-characterized molecules are Rho, which controls the stress fibers and focal adhesion formation, and Rac and Cdc42, which regulate membrane ruffling, and filopodium formation, respectively. A structural feature that distinguishes the Rho proteins from other small GTPases is the so-called Rho insert domain located between a β strand and an α helix within the small GTPase domain [1-3]. Typically Rho proteins are 190-250 residues long and consist only of the GTPase domain and short terminal C-terminal extensions. Within their GTPase domains, they share approximately 30% amino acid identity with the Ras proteins and 40-95% identity within the family. All members contain the sequence motifs characteristic of all GTP-binding proteins, bind to GDP and GTP with high affinity. In addition, the majority of members undergo C-terminal post-translational modification by isoprenoid lipids. Together with other C-terminal modifications or sequences, isoprenoid addition facilitates their subcellular location and association with specific membranes or organelles. These lipid modifications are mainly palmitoylation or prenylations, being farnesylation and geranyl-geranylation the most frequent post-translation modifications [4].

Rho GTPases work as sensitive molecular switches existing either in an inactive, GDP-bound form or an active GTP-bound form. They are endowed with GTP hydrolytic activity, mainly involved in cytoskeleton rearrangements and cell motility, but also involved in cell proliferation, transformation and differentiation [2]. Among other members, we will focus our attention on the Rac and Rho subfamilies, as they are the main effectors of cell motility.

The exchange of GDP to GTP and thus the activation of Rho GTPases is catalyzed by guanine nucleotide exchange factors (GEFs), which act downstream of numerous growth factor receptors, integrins, cytokine receptors, and cadherins. Rho GTPases are key integrating molecules from different extracellular signals, as they can be activated by different GEFs. In turn, GTP-bound active GTPases can interact with a plethora of different effectors which mediate the different cellular functions of this family of proteins. Rho GTPase effectors are a large group of proteins and include actin nucleation promoting molecules, adaptors, as well as kinases. Two factors concur to determine specific Rho GTPase function: tissue specificity of GTPase effectors and distinct intracellular localizations of closely related Rho GTPases, due to different lipid modifications [1]. The GEF family is really large, consisting of over 70 proteins mainly belonging to the Dbl or the Dock families [5,6]. Lipid modification of Rho and Rac GTPases are also strategic for subcellular compartmentalization, allowing interaction with membrane-localised GEFs upon masking of isoprenoids by GDI. The hydrolysis of GTP and contact with GAPs allows a new association of the GTPases with GDI and return to the cytosol [7]. In addition, Rho GTPases can also be regulated by phosphorylation. RhoA has been reported to be phosphorylated by protein kinase A and G (PKA and PKG) at serine at position 188, without any modification of its interaction with GEFs, but increasing its interaction with GDI and leading to extraction of RhoA from plasmamembrane [8].

Inactivation of Rho GTPases is due to an intrinsic GTPase activity, which hydrolyses GTP to GDP. However, this activity is very weak and needs to be up-regulated by GTPase activating enzymes (GAPs). Of note, Rnd1-3 [9] and RhoH [10,11] are not regulated via GAPs, due to their inability to hydrolyse GTP, and are therefore regulated through gene expression and protein degradation. An additional negative control is achieved through Rho guanine nucleotide dissociation inhibitors (GDIs). They bind Rho GTPases and prevent their activation by means of blocking interaction of the GTP-bound form with effectors, sequestering GDP-bound Rho proteins in the cytoplasm away from the GDP-GTP cycle, as well as by changing membrane compartment to GTPases [12]. Beside the GF family, the GAP group is also huge: more or less 100 members have been found in the human genome, but their regulation are even less clear than those of the GEFs. Indeed, external to their GEF or GAP domains, these proteins strongly diverge in structure and secondary functions [6,13].

The Rac-related subfamily includes Rac1 (and its splice variant Rac1b), Rac2 and Rac3 [4]. Seevaral Rac-related proteins, sharing more than 80% identity, they stimulate the formation of lamellipodia and membrane ruffles, presumably through interaction with the WAVE complex [14]. The splice variant Rac1b contains an additional C-terminal 19-residue insert and is constitutively active due to an increased intrinsic guanine nucleotide exchange rate, decreased intrinsic GTPase activity, its inability to interact with RhoGDI and enhanced association with the plasma membrane [15,16]. In addition, Rac1 can also be regulated by phosphorylation by Akt on Ser71, thereby leading to inhibit the binding of GTP but not Rac1 GTPase activity [17].

Rac1 is ubiquitously expressed, whereas Rac2 is expressed only in hematopoietic cells, where it seems to have specialized functions [18]. Rac2 inactivation has been correlated with several neutrophilic, phagocytic and lymphocytic defects [19]. Indeed, Rac2 is mainly responsible for activation of NADPH oxidase and consequent generation of reactive oxygen species (ROS) in hematopoietic cells [20]. Finally Rac3, highly expressed in brain and upregulated upon serum stimulation of fibroblasts [21], is strongly localized to the membranes where it appears to be hyperactive [22].

Animals have 3 Rho isoforms, RhoA, RhoB, and RhoC, sharing 85% amino acid sequence identity [1,6]. Despite their similarity, both modulators (GEFs and GAPs) and downstream effectors show favoured interaction with single Rho isoforms, and the three proteins play differential roles in cells. RhoA and RhoC play key roles in the regulation of actomyosin contractility and in cell locomotion, while RhoB, primarily located in endosomes, has been shown to regulate intracellular trafficking and cell survival [23]. Mostly, the functional differences are a consequence of divergence in their C-terminal 15 amino acids, where the highest level of difference is found.

### Molecular mechanism of cell migration

Cell migration in tridimensional extracellular matrix (ECM) is a multistep process involving changes in the cytoskeleton, cell-substrate adhesions and the extracellular matrix components. Cell migration is generally initiated in response to extracellular stimuli, which can be diffusible factors, signals on neighboring cells, and/or signals from the extracellular matrix. The idea that Rho family GTPases could regulate cell migration derives from observations that they mediate the formation of specific actin containing structures [24,25]. Furthermore, Rho proteins regulate several other processes relevant to cell migration, including cell-substrate adhesion, cell-cell adhesion, protein secretion, vesicle trafficking and transcription.

**Figure 1:**
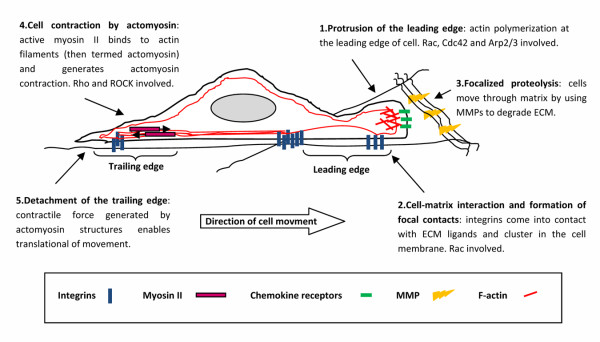
**Cell migration in 3 D matrix**. See text for detailed explanation of motility steps.

Cell migration in three-dimensional ECM can be schematized into five separate steps [26] (figure 1):

1. lamellipodium extension at the leading edge

2. formation of new focal adhesions complexes

3. secretion of surface protease to ECM contacts and focalized proteolysis

4. cell body contraction by actomyosin complexes

5. tail detachment

*Lamellipodium extension at the leading edge *involves actin polymerization, and it is known that lamellipodia consist of branched or unbranced filament networks formed through the actin-nucleating activity of the actin-related proteins 2/3 (Arp2/3) protein complex [27,28]. Rac stimulates new actin polymerization, acting on Arp2/3 complex, which binds to a family of proteins called nucleating promoting factors (for detailed description of actin nucleation factors refer to [29,30]) and initiates the formation of new actin filaments on the sides of existing filaments to form a branching actin network [27]. The Arp2/3 complex is activated by Rac through its target insulin receptor tyrosine kinase substrate p53 (IRSp53) [31]. Rac interacts with IRSp53, which in turn interacts through an Src-homologous domain 3 (SH3) domain with a member of the WASP family, which then binds to and activates the Arp2/3 complex. Rac is required for lamellipodium extension induced by growth factors, cytokines and extracellular matrix components [32]. Rac activation by both tyrosine kinases and G-protein-coupled receptors is dependent on phosphoinositol3-kinase (PI3K) activity, and inhibitors of PI3K block Rac activation [33]. During lamellipodia extension phosphoinositol phosphates (PIPs) also bind and activate GEFs that regulate the activity of Rac that bind the Arp2/3 complex[34].

A number of myosins, the main motor protein in eukaryotic non-muscle cells, have been implicated in cell migration [35]. Myosin light chain (MLC) phosphorylation is enhanced in the lamellipodial region of cells [36], which suggests a role for myosins in lamellipodium extension. Rac can affect the phosphorylation of both myosin heavy chain (MHC) [37] and MLC via activation of its downstream kinase p21 activated-kinase (PAK) [38].

*Formation of new focal adhesions complexes *is localized in the lamellipodia of most migrating cells. Upon the attachment of the extending lamellipodium to the extracellular matrix, integrins come into contact with ECM ligands and cluster in the cell membrane interacting with the focal adhesion kinase (FAK), α-actinin and talin. All these proteins can bind adaptor proteins through SH2, SH3 or proline rich domains to recruit actin binding proteins (vinculin, paxillin and α-actinin) as well as regulatory molecules PI3K to focal complexes [39,40]. Rac is required for focal complex assembly [41]and cell adhesion to the extracellular matrix itself activates Rac [42].

*Secretion of surface protease to ECM contacts and focalized proteolysis *is crucial for cells to migrate in a three-dimensional matrix and, even on a two-dimensional matrix, protease production can be important for migration [43]. There are some indications that Rho GTPases could play a role in regulating the secretion and/or activation of secreted proteases. For example, Rac is required for shear stress-induced matrix metalloproteinase 9 (MMP9) expression in chondrocytes [44], and activated Rac can induce expression of the MMP1 in fibroblasts [45]. Constitutive expression of activated Rac induces activation of Jun N-terminal kinase (JNK), which phosphorylates and activates the transcription factor Jun. Jun is a component of the activator protein 1 (AP-1) transcription factor complex and regulates transcription of many genes, including MMP genes [46]. Furthermore, in HT1080 cells, Rac1 mediates MMP2 activation and membrane type matrix metalloproteinase (MT1-MMP) expression/processing during the encounter between invading tumor cells and type I collagen-rich stroma, thereby facilitating collagenolysis and cell invasion [47].

*Cell body contraction *is dependent on actomyosin contractility. The contraction of actin filaments is provided by myosin II. Stress-fiber assembly and contraction, which are controlled by myosin II, are predominantly induced by the small G-protein Rho and its important downstream effector, the Rho-associated serine/threonine kinase (ROCK). Rho acts via ROCKs to affect MLC phosphorylation, both by inhibiting MLC phosphatase and by phosphorylating MLC [48]. It is likely that ROCKs and MLCK act in concert to regulate different aspects of cell contractility, because ROCK appears to be required for MLC phosphorylation associated with actin filaments in the cell body, whereas MLCK is required at the cell periphery [49]. This allows the cell to separately control cortical actin dynamics from contractions in inner regions.

*Tail detachment *occurs when cell-substrate linkages is preferentially disrupt in the back of the cell, whereas the leading edge remains attached to the ECM and further elongates [50]. At the trailing edge, focal complex dissassembly occurs through several mechanisms dependent on the type of cell and strength of adhesion to the extracellular matrix [51]. In slowly moving cells tail detachment appears to depend on the action of the protease calpain, which cleaves focal complex components like talin and cytoplasmic tail of β1 and β3 integrins at the rear of cells [52]. A reduction in Rho activity could inhibit tail detachment, through decreased actomyosin contractility [53].

### Diversity of tumor invasion mechanisms

A combination of in vivo imaging and 3 D in vitro models have shown that cells could move using different motility styles. Indeed cells can move as individual cells or in solid multicellular component. Single-cell migration includes mesenchymal and amoeboid migration strategies, whereas collective migration is referred to multicellular strands, sheets, cluster and cohorts. Differences in extracellular protease activities, integrin-mediated cell-matrix adhesion, cadherin-mediated cell-cell adhesion, cell polarity and cytoskeletal arrangement define the type of cell migration and invasion.

### Mesenchymal motility

Mesenchymal motility is characterised by an elongated, fibroblast-like, cell morphology with established cell-polarity and is dependent, upon proteolysis, to the degradation of the ECM [54,55]. In this kind of motility, cell speed is relatively slow (0.1-1 μm/min). Upon several stimuli, phosphatidylinositol (3,4,5)-triphosphate (PtdIns(3,4,5) *P*_3_) is generated at the leading edge of the cell and leads to cell polarization through activation of the small GTPase Rac1, which in turn organizes actin polymerization and *lamellipodium *formation [56,57]. Activation of cell division control protein 42 homolog (Cdc42) and the recruitment of adaptor proteins can also promote actin polymerisation. The directionality of cell movement is maintained by Cdc42, which coordinates actin polymerisation at the front of the cell with microtubule attachment and alignment [57,58]. Together, these events lead to the formation of an actin-rich protrusion. After the extension of the protrusion, small integrin-dependent focal complexes are formed that attach the new protrusion to the ECM. Some focal complexes then develop into large focal adhesions that enable actomyosin contractile force to be transmitted to the ECM [57]. The role of RhoA and its effectors ROCK in mesenchymal motility is complex; their activity needs to be reduced to extend protrusions at the front of the cell [59], but they promote the retraction of the lagging tail [57]. As a result, the overall effect of inhibiting these proteins in mesenchymal cells is often minimal [60]. Mesenchymal cells are able to move through a matrix-filled space by using proteases, such as MMPs and urokinase-type plasminogen activator (uPA), that degrade ECM proteins and creates the path [61,62].

### Amoeboid motility

Amoeboid movement of cells is likely to use similar mechanisms of the migration of leukocytes and *Dictyostelium discoideum *[63]. This movement is very similar to the rounded Rho- and ROCK-dependent form of motility that has been described in A375m2 melanoma and LS174T colon carcinoma [60]; With the advent of multiphoton microscopy, intravital imaging of mammalian systems has greatly improved and has opened up new ways to explore chemotaxis, cell-cell interactions and the metastatic cascade within the in vivo microenvironment. High resolution intravital imaging have demonstrated that some carcinoma cells move at very high speed with an amoeboid morphology (up to 4 μm/min) in vivo [54,64]. This motility style is largely independent from cell-ECM contact and from proteolytic degradation of ECM from MT or soluble MMPs. Amoeboid moving cells show rounded morphology and greatly exploit as a propulsory force the acto-myosin cytoskeleton contractility, without Rac-driven cell polarization, but requiring Rho activation. Cortical actin contraction driven by Rho-ROCK signalling through myosin activation might promote the rapid remodelling of the cell cortex characteristic of amoeboid movement [54,60,65].

Furthermore, cell-ECM attachments of amoeboid moving cells are not organized in large focal adhesions but are very diffuse and much weaker cell-ECM attachments are required, because amoeboid movement cannot be blocked by inhibition of integrin function [55,66]. Notably, proteases are not required for this style, because cells are able to squeeze through gaps in the ECM instead of degrading it [66]. These differences in "path generating" by proteolysis for mesenchymal moving cells or in "path finding" by squeezing for amoeboid cells could explain the different speeds of the two styles.

### Collective motility

A third form of motility is collective cell motility. Collectively migrating cells maintain their cell-cell junctions and migrate in sheets, strands, tubes and cluster, either still in connection with their originating tissue or as separated, independently migrating cluster. In cancer, collective cell migration and invasion is found in distinct cancer types, including high and intermediate differentiated types of lobular breast cancer, epithelial prostate cancer, large cell lung cancer, melanoma, rhabdomyosarcoma, and most prominently in squamous cell carcinoma. High-resolution multimodal microscopy has shown that the guiding cells use β1-integrin-mediated focal adhesions and local expression of MT1-MMP at their leading edges to cleave collagen fibers and orient them in a way that generates tube-like microtracks into which the collective mass migration of follower cells can occur [67,68]. Mechanistically, this is similar to a collective form of mesenchymal motility, with the cells at the front producing MMPs and generating a 'path' for the following cells [68]. In contrast to single cell movement, which requires the loss of adherens junctions, the maintenance of adherens junctions is important for this form of movement [67]. The mechanics of this form of motility are poorly understood because of the difficulties of modelling it *in vitro*. However, the regulatory pathways underlying collective cell migration have just begun to be elucidated and its clinical manifestations, prognostic value, and actual contribution to metastasis remain to be assessed

### Plasticity of tumor-cell migration

Cells from several origins, and among them cancer cells are particularly talented, are able to engage *ad hoc *epigenetic/ontogenetic programmes enabling them to adapt to environmental changes. This ability of cells, commonly referred as cell plasticity, is often related to different strategies to move in 3 D tissues [65,69].

Loss of epithelial-like cell morphology to adopt a motile phenotype has been termed epithelial-mesenchymal transition (EMT). This is a profound change in cell phenotype that causes immotile epithelial cells to acquire traits such as motility, invasiveness, and resistance to apoptosis or the ability to adapt to environmental changes and continue to invade successfully. These features are driven by extracellular signals, most of which are still unknown, which in turn induce expression of a series of transcription factors guiding the achievement of the new plastic, adjustable phenotype.

In the EMT process, the cells lose their epithelial characteristics, including their polarity and specialized cell-cell contacts, and acquire a mesenchymal migratory behaviour, allowing them to move away from their original site towards remote locations [70,71]. EMT illustrates the differentiation plasticity during development, but is commonly exploited by cancer cells to invade and metastatize [69,71,72]. Mesenchymal motility is characterised by elongated and polarized cell morphology; it depends upon ECM proteolysis of the moving cells which, through production of MMPs, generates a 'path'. Although several soluble factors that promote this process have been identified, the pathophysiologic context in which they act remain unclear. Inflammation is a key conspirator in the emergence of EMT in adults, although it is absent during embryonic development, suggesting the existence of multiple stimuli eliciting EMT and possible multiple different subtypes of EMT. Recently Kalluri and Weinberg proposed a classification of EMT: type 1 EMT serves for embryonic development, type 2 for tissue repair and type 3 for metastatic spreading of cancer [71]. While type 1 EMT is independent from inflammation and injuries, both type 2 and 3 share their dependence from inflammation and are characterized from their endurance until the provoking spur is removed. Of note, exogenous addition of MMP-3, MMP-2 or MMP-9 facilitate EMT likely through cleavage of E-cadherin [73,74]. Finally, type 3 EMT is facilitated by genomic and epigenetic alterations acquired by cancer cells, and some of these alterations have been reported also in tumor-associated stroma.

The EMT transcriptional programme has been associated with activation of several key transcriptions factors, including Snail-1 and Snail-2 (Slug), Twist, ZEB-1-2, etc. The large number of transcription factors which can be engaged to elicit the same phenotype, i. e. the transition from an epithelial-like to a mesenchymal-like cell behaviour, is not necessary indicative of redundancy. Indeed, different stimuli able to elicit EMT appear to act on different transcription factors. Tumor microenvironmental cues as inflammation [75] or stromal fibroblasts (Chiarugi, P., unpublished data) drive a Snail-1-dependent EMT, while intratumoral hypoxia elicits a Twist-mediated EMT [76-78]. In addition, miRNAs are able to regulate EMT acting mainly on ZEB-1 and ZEB-2 [79,80]. The transcriptional programme leads to regulation of a series of proteins: decrease of E-cadherin for disruption of adherens junctions, increase in N-cadherin and Met proto-oncogene to drive motility, as well as increase in MMPs and uPA/uPAR proteolytic systems to degrade 3 D barriers [71,72].

In response to particular environmental cues, cancer cells can *de novo *acquire an amoeboid-like motility, thus undergoing to what has been termed mesenchymal to amoeboid transition (MAT). The latter is a primitive form of cell migration that allows cells to glide through, rather than degrade, ECM barriers through weakened cell-ECM attachments. Conversely to mesenchymal motility, cells moving through an amoeboid mode show independence from proteolytic systems to degrade 3 D barrier and the movement of cells depends on their ability to squeeze between gaps of ECM instead from the ability to degrade it [55,65].

MAT can be induced in cells by both environmental or epigenetic cues. In fibrosarcoma and melanoma cells the inhibition of integrin or MMP function, leads to switch from mesenchymal to an amoeboid-like migration program, thereby rescuing motility by alternative mechanisms and sustaining the dissemination of single cancer cells [55,66,81]. Indeed fibrosarcoma and melanoma cells, in the presence of a cocktail of a broad spectrum protease inhibitors, convert their motility style from proteolytic to amoeboid, thus undergoing MAT. In keeping with the different dependence of mesenchymal or amoeboid motilities from integrin engagement, treatment of sarcoma cells with integrin antagonists elicits a clear MAT [55,66,81,82].

Beside environmental regulation, MAT can also be induced by epigenetic expression of regulating factors. First, prostate carcinoma cells move through and EphA2-mediated amoeboid motility [83,84]. Second, aggressive melanoma cells are able to shift *ad hoc *between mesenchymal and amoeboid motility: in response to pro-inflammatory cytokines they undergo EMT, while after re-expression of embryonic EphA2 receptor, experience a new kind of motility program undergoing MAT [81]. In addition, fibrosarcoma cells have been reported to undergo MAT during forced activation of stathmin, a known microtubule cytoskeleton regulator, or during inhibition of the E3-ubiquitin ligase for RhoA Smurf1 [85,86]. Finally, loss of p53 or p27 tumor suppressors promotes RhoA/ROCK-dependent cell migration and invasion in 3 D matrices for human melanoma cells, suggesting that MAT is associated with worse prognosis cancers [87-89].

Nevertheless several interesting data indicate that MAT is an efficient plasticity programme for cell motility, the identification of the molecular players regulating MAT is still at its infancy. In any case, as the different reported examples of MAT share some key features, as cell body constriction and independence from proteases, we speculate that MAT, as well as EMT, should be driven by a transcriptional response. One the first event could be the repression of EMT, i.e. an "inverse" transcriptional programme", and of its transcriptional executors Snail and Twist, but it is likely that MAT switches on its own transcription factors.

Similarly with respect to EMT, the transition from collective invasion to amoeboid movement relies in weakening cell-cell and cell-ECM interactions, i. e. disruption of E-cadherin mediated adherens junction and integrin-linked focal complexes [68,90]. Melanoma cells have been indicated to move in cohorts of multicellular clusters but the contextual inhibition of β1 integrins abolished these collective movement, thereby inducing detachment of individual single moving cells using amoeboid style to invade, i.e a collective to amoeboid transition (CAT) [91]. To date it is unknown that CAT converts collective migration to the amoeboid one directly or via an intermediate mesenchymal migration step [55].

### Reciprocal control of Rac and Rho small GTPases

A mutual antagonism between the Rac and Rho GTPases has been observed in several cellular settings, raising the significant question of its integrated in cell behaviour. In A375M2 melanoma cells, displaying a predominantly amoeboid phenotype with a minority of cells migrating in a mesenchymal fashion, Sanz-Moreno identified DOCK3 as a GEF specific for Rac1, NEDD9 as an adaptor protein of the p130Cas family binding DOCK3, and WAVE2 as a protein that promotes actin nucleation downstream of Rac [92]. In this cell model there is a reciprocal inhibitory relationship between Rac and Rho signaling cascades establishing a regulatory switch between the mesenchymal and amoeboid phenotypes. Mesenchymal melanoma morphology and invasiveness style is controlled by a Rac1 activation pathway, mediated by adaptor protein NEDD9 and DOCK3, acting as a Rac1-GEF. Cell elongation and actin polymerization downstream to Rac1 is mediated by the actin-nucleation protein WAVE2. WAVE2 is also responsible for downregulation of actomyosin contractility, cytoplasm blebbing and amoeboid motility. On the contrary in amoeboid moving cells, Rho activation stimulates a ROCK-mediated actomyosin and cell body contractility. In parallel, amoeboid signalling leads to downregulation of mesenchymal movement, mainly through inhibition of Rac1 by activating the Rac-GAP ARHGAP22, thereby completing the circuitry of Rac1-RhoA antagonism.

Beside ARHGAP22 and DOCK3/NEDD9 signaling, other pathways leading to shift of Rac/Rho balance in favor of the last induce MAT as well. These include the interference with Rab5-mediated endocytosis and recycling of Rac to cell protrusions [93] and the inhibition of E3 ubiquitin ligase Smurf1, which leads to Rho degradation directly at the leading edge and thereby grants for dominance of Rac at the front of polarized cells [86].

**Figure 2:**
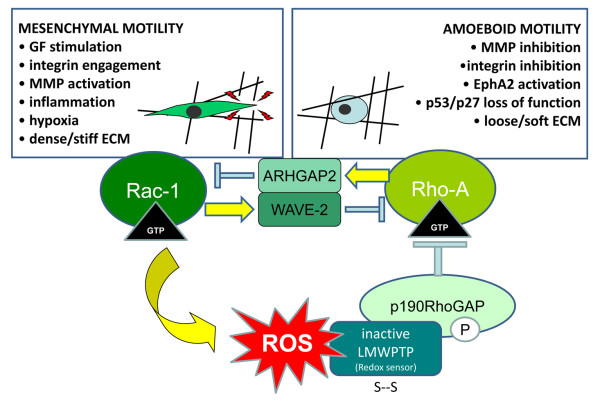
**Reciprocal regulation between Rho and Rac during mesenchymal or amoeboid motility styles**. ROS act as a balance for Rac-1/RhoA antagonism. Indeed Rac-1, which drives oriented mesenchymal motility, leading edge protrusion and lamellipodia formation, is a key molecular player of regulated intracellular ROS sources. Rho activation is responsible for amoeboid motility, a non-oriented movement which enables the cell to squeeze between gaps of ECM instead of proteolytically degrade it. Hence, upon Rac activation oxidation/inactivation of the LMW-PTP which normally activate the Rho regulator p190Rho-GTPase, leads to RhoA down-regulation. Conversely, low ROS intracellular content lead to RhoA activation, through LMWPTP activation and p190RhoGAP dephosphorylation/inactivation. Activated RhoA is able to inhibit Rac-1 through the ARHGAP2 (also named chimerin-2), while Rac1 activates WAVE2 which in turn inhibits RhoA.

Studies on specific extracellular signals acting in favour of mesenchymal or amoeboid movements are still at their infancy. Clearly, extracellular factors act on different invasive styles through Rac and Rho modulation and the role of their GEFs and GAPs is mandatory. Presumably both Rac1 and Rho activation are ultimately controlled by GFs and integrin activity, thereby suggesting the existence of additional mechanisms by which Rac can inhibit the Rho-mediated amoeboid phenotype. Activated Rac in response to integrin engagement has been shown to stimulate the activity of p190RhoGAP (which down-regulates the activity of Rho isoforms) by promoting its phosphorylation. Indeed, the oxidative cascade involving Rac1, reactive oxygen species (ROS) and a p190RhoGAP phosphatase has been correlated with the antagonistic crosstalk between Rac1 and the RhoA [94]. Extracellular activation of Rac1 leads to enhancement of ROS production and this leads in turn to redox inhibition of Low-Mw protein tyrosine phosphatase (LMW-PTP), finally enhancing the phosphorylation of its substrate p190RhoGAP [94,95]. The redox circuitry engaged by mesenchymal stimuli is closed because phosphorylated p190RhoGAP downregulates RhoA and suppresses amoeboid activity. In a specular fashion, activation of the repulsive EphA2 receptor in prostate carcinoma cells is accompanied by reduced Rac1 activity and attenuated generation of ROS, which leads to LMW-PTP activation, p190RhoGAP dephosphorylation and to an increase of Rho signaling [83,96]. EphA2 receptor activation by its cognate ligand ephrinA1 is a powerful signal to activate RhoA and its overexpression and activation causes achievement of amoeboid invasive styles from both prostate carcinoma and melanoma cells [81,83,84,96]. In addition, p120-catenin supports Rac-Rho crosstalk by controlling the cortical localization of p190RhoGAP and thereby allowing Rho inhibition through activation of Rac [97,98]. Indeed, in p120-deficient cells, p190RhoGAP was activated via its redox pathways by a constitutively active Rac mutant but was unable to inhibit Rho [97]. This coordinated and opposed activity of Rac1 and RhoA is crucial to cellular dynamics, the former promoting membrane protrusion, cell polarity and spreading, the second cytoskeleton contractility and tail retraction Collectively, the above evidence indicate that the intricate cross-talk between Rho family GTPase that underlies dynamic cell responses is in large part redox regulated (figure 2).

Moreover, Radisky et al. reported that activation of EMT in breast carcinoma cells is associated with expression of the alternatively spliced Rac1 isoform, the constitutively active Rac1b, and thereby to ROS generation. Although the identification of the direct redox protein sensors driving EMT is still lacking, these oxidant have been proved to be genotoxic, thereby contributing to both carcinogenesis and tumor invasiveness [74]. Our preliminary observations in this context indicate that in cancer cells the ROS generated upon EMT commitment also retain signalling roles, enhancing expression of transcription factors correlated with mesenchymal or inflammatory programs as Snail-1, Twist, hypoxia inducible factor-1 or nuclear factor κ-B (Chiarugi, P., unpublished results). In the context of EMT the downregulation of Rho proteins, although highly feasible, remains to be described in its molecular details.

### Aberrant regulation of Rac and Rho proteins in cancer

**Figure 3:**
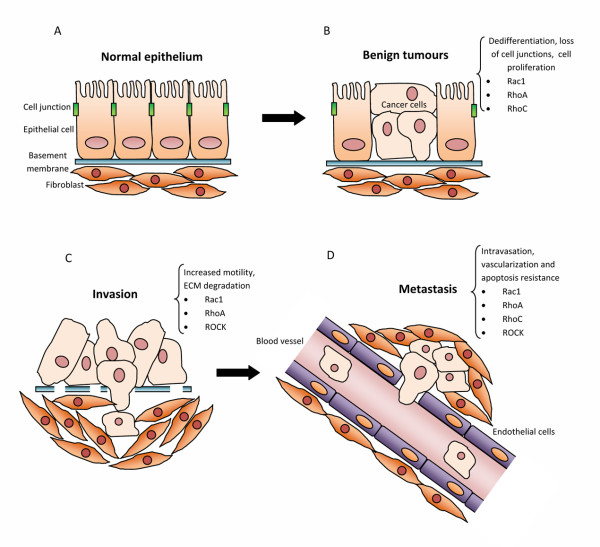
**Involvement of Rho proteins at different stages of tumor progression**. A) Maintenance of normal epithelial cell polarity. B) Benign tumors: once a tumor is initiated, Rho proteins can contribute to tumor development by stimulating dedifferentiation, growth and loss of cell polarity. C) Locally invasive tumors: Rho proteins can contribute to tumor development by altering cell-cell and cell-matrix adhesion, Rho proteins allow tumor cells to become invasive. D) Metastasis to distant site: Rho and ROCK are required for tumor cells to cross endothelial cell layers. RhoC promotes expression of angiogenic factors, leading to an increase in vascularization of the tumor.

As a consequence of the large number of key functions assigned to Rho proteins, like proliferation, apoptosis/survival, cell polarity, cell adhesion and plasticity of cell migration [99,100], it is not surprising that they play important roles in tumor biology [101]. A clear connection can be established between Rho proteins overexpression and a large variety of human tumors [102,103]. Rho GTPases have been reported to contribute to most steps of cancer initiation and progression including the acquisition of unlimited proliferation potential, survival and evasion from apoptosis, angiogenesis, tissue invasion and the establishment of metastases (figure 3). Some Rho GTPases stimulate cell cycle progression and regulate gene transcription, and this could in part explain their pro-oncogenic properties, for example in promoting Ras-induced transformation [104]. Some Rho GTPases are thought to be able to regulate the release of pro-angiogenic factors to promote neovascularisation [105]. The ability of Rho GTPase family members to regulate loosening of epithelial cell-cell contacts, MMPs expression and the plasticity of cell migration (EMT, MAT) [103] points to a central role in cancer cell invasion and metastasis (figure 3).

Primary tumors generally arise as a consequence of multiple mutations and epigenetic changes affecting key genes that ultimately affect proliferation and survival. Unexpectedly, to date, no mutations have been found in Rho proteins. Only one member of the Rho family of small GTPases (RhoH) has been reported to be genetically altered in non-Hodgkin's lymphomas and multiple myeloma. Since mutations in Rho proteins have not been found, deregulation of Rho GTPase signalling could occur at the level of expression or activation of Rho GTPases, accomplished by the level of expression or activation of their regulators or downstream effectors. RhoA and RhoC expression and/or activity is frequently increased in human tumors, whereas RhoB is often downregulated [106]. Increased RhoA expression was described in various human tumors including liver [107], skin [108]and colon cancer [109]. In liver, increased RhoA expression correlated with increased RhoA activity, poor prognosis and recurrence [107]. Elevated RhoA levels also corresponded to progression of ovarian [110], bladder [111], gastric [112] esophageal squamous cell [113], and testicular cancer [114] (table 1). RhoA has been implicated in virtually all stages of cancer progression: for example, in vitro, constitutively active RhoA can stimulate transformation [115]. In normal epithelia, RhoA contributes to the generation of epithelial polarity and junction assembly and function [116] but also affects epithelial disruption during tumor progression. RhoA activity can be inhibited downstream of cadherins leading to a more motile phenotype [97]. Different GEFs and GAPs influence how Rho proteins can act in different contexts either promoting epithelial organization and polarity or epithelial EMT, as seen in studies of RhoA GEFs/GAPs in Drosophila models [117].

**Table 1:** Aberrant regulation of Rho proteins in cancer.

Rho proteins	Mechanism of deregulation	Tumor type
**RhoA**	High protein levels, high signalling activity	Liver [107], skin [108], colon [109], ovarian [110], bladder [111], gastric [112], esophageal squamous cell (SCC) [113], testicular [114], breast [109]

**RhoB**	Overexpression or downregulation	Breast (overexpression) [109], lung (downregulation) [151]

**RhoC**	High protein levels, high signalling activity	Melanoma metastases [119], breast [121], squamous cell (SCC) [122], pancreas [123], liver [124], ovarian [110], head and neck [120], prostate [126], non-small cell lung (NSCLC) [127], gastric cancer [125]

**RhoH**	Rearrangement and mutations (5' UTR)	non-Hodgkin's lymphomas and multiple myeloma [152]

**Rac1**	High protein levels, high signalling activity	Testicular, [114]gastric [112], breast [136], squamous cell (SCC) [137]

**Rac1B**	Alternative splicing	Colon [148]; Breast [136]

**Rac2**	High protein levels	Head [108] neck squamous-cell carcinoma (SCC) [122]

**Rac3**	Hyperactive or overexpression	Breast [22]

A 3 D in vitro invasion model using co-cultures of SSC12 carcinoma cells with stromal fibroblasts, has shown how this fibroblast generates the traction force and remodels the matrix through MMPs. Different Rho GTPases were found to be required in the leading fibroblast and the following carcinoma cells with a RhoA regulation of MLC in the former and mainly Cdc42 and myotonic dystrophy kinase-related Cdc42-binding kinase (MRCK) function in the latter [118]. In contrast to RhoA, RhoC has no apparent transforming activity. RhoC was identified in a screen for genes upregulated in melanoma metastases [119], and has subsequently been proposed as a marker for poor prognosis in cancers of different origins [120]. RhoC is upregulated in many cancers including breast cancer [121] and squamous cell carcinoma (SCC) of skin [122] (table 1). Increased expression of RhoC correlates with progression and poor prognosis of ductal adenocarcinoma of pancreas [123], hepatocellular cancer [124], breast cancer [109], ovarian cancer [110], bladder cancer [111], gastric cancer [125], esophageal SCC [113], head and neck SCC [120], prostate cancer [126], and non-small cell lung carcinoma (NSCLC) [127]. In contrast to RhoC, expression of RhoA and RhoB did not correlate with poor prognosis in pancreatic cancer [123]. Increased RhoC expression has been claimed as the possible cause for the induction in invasion and metastasis triggered by the overexpression of the microRNA-10b in breast cancer [128]. RhoC expression is increased during EMT in a colon cancer model and contributes to EMT-induced migration, whereas RhoA levels go down [129]. It is not yet clear how RhoC increases invasion and metastasis or why its effects differ from RhoA. Some reports indicate that RhoA, RhoC and their downstream target ROCK are needed for cancer cell extravasation [130]. Interestingly, RhoC can induce the production of angiogenic factors in breast cancer, and this could help promote entry into blood vessels and thereby metastasis dissemination [105]. Unlike RhoA and RhoC, RhoB is often downregulated in human tumors and its expression inversely correlates with tumor aggressiveness. It has been proposed that RhoB can work as a tumor suppressor as it is activated in response to several stress stimuli including DNA damage or hypoxia, and it has been reported to inhibit tumor growth, cell migration and invasion and have proapoptotic functions in cells [131]. RhoB knock-out mice develop normally but have enhanced carcinogen-induced skin tumor formation, in agreement with a role of RhoB as a tumor suppressor [132]. RhoB also suppresses invasion: for example it has been postulated to act downstream of protein kinase C in the regulation of cancer cell invasion in vitro [133] and it was also reported to inhibit Ras-induced invasion and metastasis [134]. The exact mechanism whereby RhoB suppresses tumor growth and invasion is not clear, although its role in endosomal trafficking could be important. RhoB regulates the delivery of signalling proteins, including growth factor receptors and the tyrosine kinase Src, to specific intracellular compartments [135], and this could certainly influence proliferation and invasion.

The Rac subfamily of Rho GTPases includes Rac1, Rac2, Rac3. Rac1 is over-expressed in various tumors and accumulating evidence indicate that Rac1-dependent cell signalling is important for malignant transformation [106]. Overexpression of Rac1 correlates with progression of testicular [114], gastric [112], and breast cancer [136]. Rac1 is also overexpressed in oral SCC [137] (table 1). Rac1 knock-out in mice is embryonic lethal [138] but conditional knock-out mice have been studied extensively [139]. In a conditional lung cancer mouse model Rac1 function was required for K-Ras-driven proliferation and tumorigenicity [140]. Similarly, mice lacking the Rac-specific GEF Tiam1 are protected from Ras-induced skin cancer, developing fewer tumors, although the tumors that do form are more aggressive [141]. These results suggest that Rac proteins normally stimulate tumor cell proliferation but inhibit tumor dissemination. Rac1 could contribute to cancer cell proliferation via regulation of the cell cycle: for example, it stimulates expression of cyclin D1, and induces cell transformation in vitro [104]. Active Rac can mediate the loss of adherens junction in some situations, promoting a more invasive phenotype [142]. Rac1 can also contribute to cancer cell invasion by regulating the production of MMPs and their natural inhibitors, the tissue-specific inhibitors of MMP (TIMPs) [143]. Like Rac1, Rac2 and Rac3 are over-expressed in some tumors. Rac3 is hyperactive and/or deregulated in breast cancers [22]. The contribution of different Rac isoforms to migration is likely to depend on the cell type and their relative expression levels. Rac2 is required for neutrophil migration but whether it acts similarly in tumors is not known [144]. In contrast, Rac1 and Rac2 are dispensable for cell migration in macrophages, although Rac1 is required for invasion [145]. Studies of Rac3-null mice indicate that Rac3 but not Rac1 or Rac2 specifically contributes to the development of Brc/Abl-induced lymphomas in vivo [146]. However, in fibroblasts, Rac1 but not Rac3 suppression by RNAi affects lamellipodium formation although cell invasion is reduced in both cases [147]. It is not yet clear how these results can be translated to cancer cell invasion in vivo.

The splice variant of Rac1, Rac1b, was initially identified to be up-regulated in colon cancers [148]. It does not bind RhoGDI and thus is present predominantly in the GTP-bound state. Although Rac1b is defective in activating several Rac1-regulated signaling pathways, in some cell types it stimulates cell survival and cell cycle progression through nuclear factor-kappa B, and is less susceptible to ubiquitination and degradation, which could explain its increased expression in cancers [149].

Rho GTPases are involved in all stages during cancer progression (figure 3). Although their initial discovery as regulators of cytoskeleton dynamics implied that they are most likely to contribute to cancer cell migration and invasion, it is now clear that the function of Rho GTPases is not restricted to these events and that they can affect tumor cells through modulation of gene transcription, cell division and survival, intracellular transport of signalling molecules or modifying the interaction of cancer cells with surrounding stromal cells. This makes the detailed analysis of how Rho GTPases work in cells and contribute to tumors very complex but at the same time promising for potential future therapeutical intervention. The involvement of specific GEFs or GAPs in defined processes regulated by Rho GTPases makes them particularly suitable as therapeutic targets [150].

## Conclusions

Metastasis is a multistage process needing a strong adaptability of cells to the different microenvironments within primary tumors, in the ECM, in blood or lymphatic streams and finally in the metastatic niche. Intravital imaging of GFP-expressing cancer cells in subcutaneous tumors illustrated this adaptability. In the core of the tumor neoplastic cells mainly moved using mesenchymal and elongated style, while cells at the tumor edge escape the tumor limit and enter the ECM using a rounded/amoeboid motility [92]. Genetic or pharmacological treatment of ARHGAP22 or ROCK shift one motility style to an other, thereby confirming the key role of Rac and Rho GTPases in plasticity of cell motility. More intriguingly, the combined treatments aimed at blocking simultaneously both modes of migration strongly inhibit the opportunistic behaviour of cancer cells, thereby limiting their invasive potential. These data indicate that the winning strategy to combat successful metastatic diffusion of aggressive cancer cells is either a combinatory treatment targeting both invasive styles, or the identification of single molecular targets driving the ability of cancer cells to adapt to environmental changes, i. e. cell plasticity itself. Unfortunately, the identification of the molecular mediators of plasticity in cell motility is still at its infancy, but it will be surely the next challenge to really target the opportunistic motility of cancer cells [69].

## Competing interests

The authors declare that they have no competing interests.

## Authors' contributions

PC and MP organized, wrote and edited the manuscript together. Both authors red and approved the final manuscript.
